# A computational analysis of atrial fibrillation effects on coronary perfusion across the different myocardial layers

**DOI:** 10.1038/s41598-022-04897-6

**Published:** 2022-01-17

**Authors:** Andrea Saglietto, Matteo Fois, Luca Ridolfi, Gaetano Maria De Ferrari, Matteo Anselmino, Stefania Scarsoglio

**Affiliations:** 1 Division of Cardiology, “Città della Salute e della Scienza di Torino” Hospital, Department of Medical Sciences, University of Turin, C.so Dogliotti 14, Turin, Italy; 2 Department of Mechanical and Aerospace Engineering, Politecnico di Torino, Turin, Italy; 3 Department of Environmental, Land and Infrastructure Engineering, Politecnico di Torino, Turin, Italy

**Keywords:** Cardiology, Mathematics and computing

## Abstract

Patients with atrial fibrillation (AF) may present ischemic chest pain in the absence of classical obstructive coronary disease. Among the possible causes, the direct hemodynamic effect exerted by the irregular arrhythmia has not been studied in detail. We performed a computational fluid dynamics analysis by means of a 1D-0D multiscale model of the entire human cardiovascular system, enriched by a detailed mathematical modeling of the coronary arteries and their downstream distal microcirculatory districts (subepicardial, midwall and subendocardial layers). Three mean ventricular rates were simulated (75, 100, 125 bpm) in both sinus rhythm (SR) and atrial fibrillation, and an inter-layer and inter-frequency analysis was conducted focusing on the ratio between mean beat-to-beat blood flow in AF compared to SR. Our results show that AF exerts direct hemodynamic consequences on the coronary microcirculation, causing a reduction in microvascular coronary flow particularly at higher ventricular rates; the most prominent reduction was seen in the subendocardial layers perfused by left coronary arteries (left anterior descending and left circumflex arteries).

## Introduction

Atrial fibrillation (AF) is the most common sustained clinical arrhythmia, strongly associated with increasing age and comorbidities^1^. Its prevalence has constantly grown during the last decades, reaching a threefold increase over the last 50 years^2^, and recent epidemiological predictions foresee a further increase, with an estimation of 16–17 million prevalent cases by 2050 in US and Europe, respectively^3^. Given this important epidemiological burden, detailed comprehension of the physiopathology of this arrhythmia is warranted.

Among the consequences of AF, patients with ongoing arrhythmia may present ischemic chest pain, ECG abnormalities (ST depression) and troponin release, even in the absence of classical obstructive epicardial coronary disease^4–6^. A possible mechanism behind this association is AF-induced microvascular dysfunction, determining a blunted coronary flow reserve even in presence of normal epicardial coronary arteries^4,7^. A direct detrimental hemodynamic effect of the irregular AF RR intervals^8–11^, however, has to date not been extensively explored as plausible alternative, or additional, mechanism^12^. Given the frequent coexistence of AF and coronary artery disease (CAD)^13^ and the lack of solid clinical data regarding the prognostic effect of AF in patients with stable CAD^14^, evaluating the rhythm-specific impact on the coronary circle appears clinically relevant.

Computational modeling in cardiology is a growing field of research, progressively used in different contexts, such as cardiac electrophysiology^15^ and cardiovascular fluid dynamics^16,17^. Mathematical modeling is a powerful tool to study the complex process of fluid dynamics, complementing empirical findings and providing quantitative insights into physiological and pathophysiological aspects of the cardiovascular system. In this respect, a recent computational multiscale model of the coronary circulation has demonstrated that AF exerts, especially at higher ventricular rates, direct epicardial coronary flow impairment, as well as an imbalance of the oxygen supply–demand ratio^18^.

By the use of an advanced computational model of the human cardiovascular system, including both arterial and venous vascular compartments, cardio-pulmonary circulation and short-term autoregulation mechanisms, aim of the present work is to deepen comprehension of coronary perfusion of the different layers of the myocardium during AF.

## Methods

Numerical simulations were carried out by means of a closed loop 1D-0D multiscale model of the entire human cardiovascular system. This computational tool is the result of several studies^19–21^, and, since its first introduction^19^, has been enriched and validated^21^. The present model merges the cardiovascular description as adopted in ^20,21^ with the coronary modeling presented in ^22,23^. A brief overview of the resulting cardiovascular-coronary framework is recalled hereunder, while more details (including full parameter settings) are offered in the Supplementary Information.

**Figure 1:**
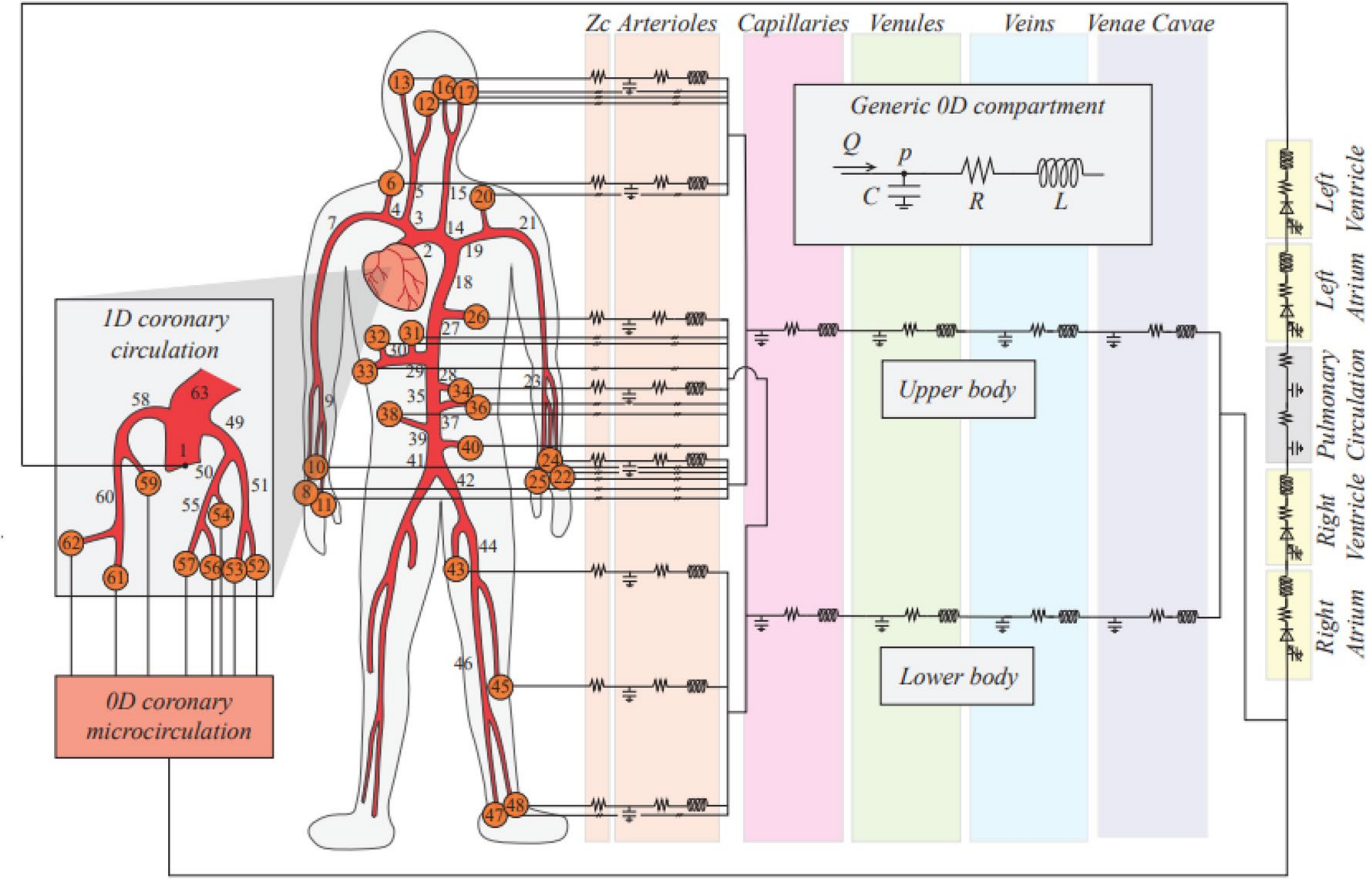
Multiscale illustration of the overall closed-loop model, with emphasis onto the coronary circulation (in the left side panel). For symmetry reasons, lower body 1D arteries were modeled for one leg only, but their contribution was doubled to account also for the other leg. Parameter setting can be found in our previous works^20,21^.

The model is composed by a 1D representation of the arterial tree, from the aortic valve to the peripheral circulation, through bifurcations and branches (accounting for 63 main vessels, Fig. 1 and Supplementary Table 1). The mathematical description of the arterial model is based on the 1D Navier–Stokes equations for mass and momentum balance. The hemodynamic variables involved are the vessel lumen area *A*, and blood flow rate *Q*. To close the system, a non-linear constitutive equation linking blood pressure *p* and area *A* is included. This relationship allows to mimic arterial viscoelastic properties and compliance. While defining initial geometry, arterial tapering is also accounted for. Blood is assumed as Newtonian, and a standard flat-parabolic profile is adopted for vessels’ cross-sectional velocity distribution. Boundary conditions at the aortic inlet and onto each terminal branch section derive from the adjacent 0D models, whereas at arterial bifurcations mass and total pressure conservation are imposed.

Each 1D terminal artery is coupled with a lumped parameter representation of the downstream circulation, as depicted in Fig. 1. This includes a lumped parameter representation of an arteriolar compartment for each 1D terminal artery (orange circles in Fig. 1), merging into two comprehensive capillary districts, according to an *upper body* and *lower body* division. Then, for both regions, the *venous return* accounts for the venular, venous and vena cava districts. The lumped models are the result of an electric-hydraulic analogy for blood pressure *p*, blood volume *V* and flow rate *Q*. Finally, the system is closed via a 0D representation of arterial and venous pulmonary circulation. The four heart chambers are contractile and modeled as separate lumped parameter compartments via a time varying elastance approach. Cardiac valves are accurately described accounting for geometric, inertial, viscous and downstream vortex effects.

The model also includes a short-term baroreflex control mechanism accounting for the inotropic effect of both ventricles, as well as the control of the systemic vasculature (peripheral arterial and capillary resistances, unstressed volume of the venous system, and venous compliance).

**Figure 2:**
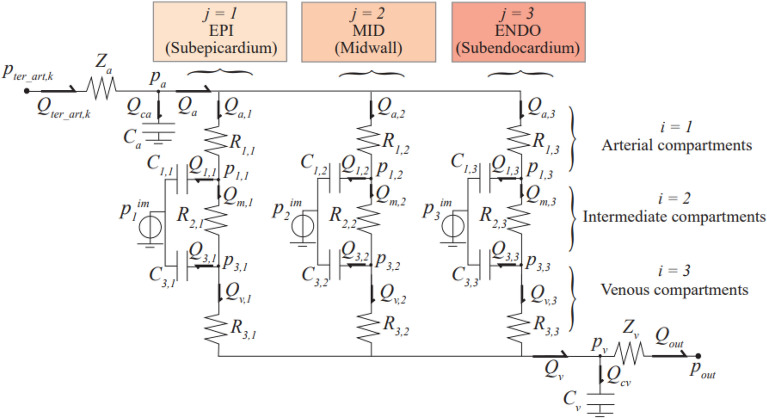
The 0D lumped parameter circuital scheme of a *k*th generic coronary distal circulation and microvasculature. The scheme allows for the subdivision among myocardial layers (index *j*) and compartments (index *i*). Acronyms *EPI*, *MID* and *ENDO* stand for subepicardium, midwall and subendocardium, respectively, whereas an arterial, an intermediate and a venous compartment may be identified. *Z*_*ca*_ and *Z*_*cv*_ represent input and output impedances of each district, *R*_*i,j*_ and *C*_*i,j*_ are the resistance and compliance parameter assigned to each *i*-th compartment belonging to the *j*-th layer, and *p*_*j*_^*im*^ is the external forcing intra-myocardial pressure. *Q*_*ter_art,k*_*, Q*_*out*_*, Q*_*a*_*, Q*_*v*_*, Q*_*ca*_*, Q*_*cv*_*, Q*_*ja*_*, Q*_*jm*_*, Q*_*jv*_*, Q*_*ij*_*, p*_*ter_art,k*_*, p*_*out*_*, p*_*a*_*, p*_*v*_ and *p*_*ij*_ are the hemodynamic variables associated with each single branch or node of the considered coronary district.

Multiscale coronary arteries and microvasculature were modeled as proposed by Mynard and Smolich^23^. The main coronary arteries (numbered 49–62 in Fig. 1) were treated as 1D vessels. Each 1D coronary artery terminates with a lumped parameter model of the downstream penetrating vasculature and microcirculatory districts, as illustrated in Fig. 2 and on the left side of Fig. 1. Such circuital representation—firstly devised by Bruinsma et al.^24^ and Spaan et al.^25^ and organized as detailed in Mynard et al.^22^, Mynard & Smolich^23^—provides a distinctive identification of the vasculature perfusing each myocardial layer, from the subepicardial (EPI), through the intermediate midwall (MID), up to the subendocardial (ENDO) circulation. These may further be subdivided in an arterial, an intermediate and a venous compartment. Resistances attributed to each myocardial layer are non-linear, depending on the current blood volume of the vessel, according to Poiseuille’s law. Compliances allow for communication between intravascular and extravascular environment, by exerting intra-myocardial pressure directly onto each myocardial layer, depending upon their relative proximity to the underlying heart chamber (the left or right ventricle cavity-induced extracellular pressure) and the associated cardiac contractility (shortening-induced intracellular pressure). The coronary loop is finally closed by connecting directly to the right atrium.

The model was employed to inquire into coronary hemodynamics during physiological (sinus) heart rhythm (SR) and atrial fibrillation (AF). We focused on three mean ventricular rates: 75, 100 and 125 bpm, which were externally imposed as inputs of the present model. To allow proper comparison between SR and AF, the model assumes resting conditions (i.e., not exercise), thus the simulations reproduce AF with variable ventricular response and SR/tachycardic right atrial pacing in non-exercising patients.

The RR beating extraction procedure is described in our previous works^18,26^. In brief, the SR beating series are pink-correlated and extracted from a Gaussian distribution with mean value *µ* = 60*/HR* and standard deviation *σ* = *cv·µ* (*cv* = 0.07 for SR, where cv is the coefficient of variation). Differently, the AF RR beating series are uncorrelated and distributed as a Gaussian function with an exponential correction with rate parameter^27^ *γ* (*cv* = 0.24 for AF). Additionally, during AF the *atrial kick* has been removed^26^ from both right and left atria, by imposing no contraction to their time-varying elastance function. For each case study, a number *N*_B_ = 2000 of RR beatings were simulated, as shown in Supplementary Fig. 1. Supplementary Fig. 2 reports a scheme of the input and output model variables.

**Figure 3:**
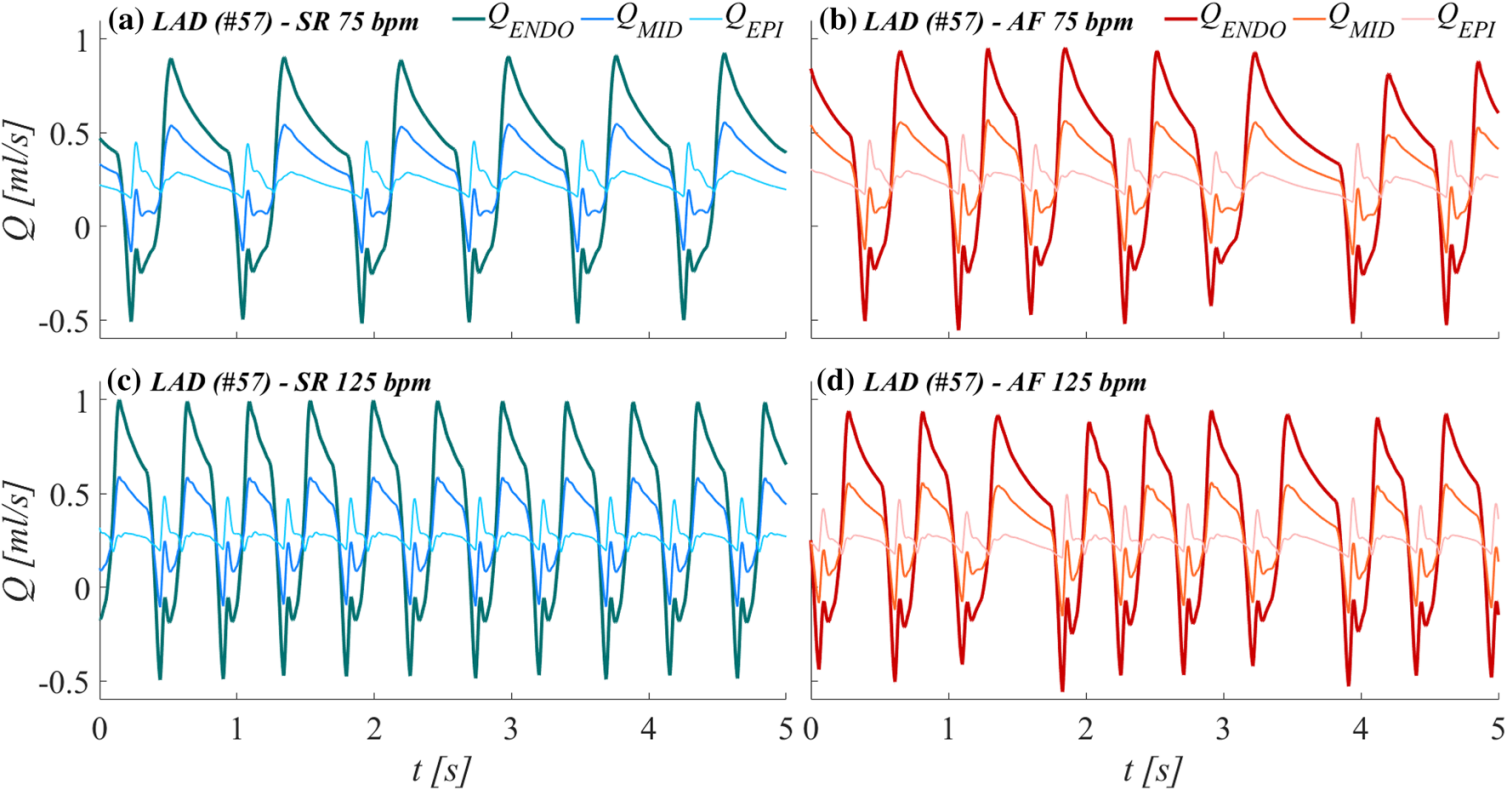
Examples of flow rate time-series *Q(t)* for the three myocardial layers (EPI: subepicardium; MID: midwall; ENDO: subendocardium) of the LAD coronary microvascular district (vessel #57). **(a)** SR at 75 bpm; **(b)** AF at 75 bpm; **(c)** SR at 125 bpm; **(d)** AF at 125 bpm.

We focused on the microcirculatory districts representing the natural termination of the 1D epicardial arteries: vessel #57 for the Left Anterior Descending Artery (LAD), vessel #53 for the Left Circumflex Artery (LCx), and vessel #61 for the Right Coronary Artery (RCA), as shown in Fig. 1. Attention was primarily addressed to the coronary arterial blood flow time series *Q*(*t*) pertaining to each myocardial layer (EPI, MID and ENDO)—denoted as *Q*_a,1_(*t*), *Q*_a,2_(*t*) and *Q*_*a*,3_(*t*) (see Fig. 2)—under both sinus and fibrillated rhythm (subscripts SR and AF, respectively). Representative flow rate time series *Q*(*t*) of the three myocardial layers pertaining to the LAD coronary microvascular district are shown in Fig. 3, in both SR and AF conditions for HR = 75 and 125 bpm.

For a generic myocardial layer and ventricular rate, beat-to-beat averaged distributions ${Q}_{SR,b}$ and ${Q}_{AF,b}$ (sized *N*_B_) were computed by averaging time series over each cardiac cycle:

$${Q}_{SR,b}=\frac{1}{{RR}_{SR,b}}{\int }_{{RR}_{SR,b}}{\mathcal{Q}}_{SR}\left(t\right)dt \,\,\mathrm{and}\,\, {Q}_{AF,b}=\frac{1}{{RR}_{AF,b}}{\int }_{{RR}_{AF,b}}{\mathcal{Q}}_{AF}\left(t\right)dt$$

Subscript *b* indicates the *b*th mean flow rate obtained from the *b*th cardiac cycle (*RR*_*SR,b*_ or *RR*_*AF,b*_ depending on the current cardiac rhythm).

A set of 9 beat-to-beat distributions of flow rate variables ${Q}_{SR,b}$ and ${Q}_{AF,b}$ was collected for each cardiac rhythm (3 myocardial layers upon the 3 mean heart rates.). Mean values ${\overline{Q} }_{SR}$ and ${\overline{Q} }_{AF}$ were calculated from these distributions as:

$${\overline{Q} }_{SR}=\frac{1}{{N}_{B}}{\sum }_{b=1}^{{N}_{B}}{Q}_{SR,b}\,\, \mathrm{and}\,\, {\overline{Q} }_{AF}=\frac{1}{{N}_{B}}{\sum }_{b=1}^{{N}_{B}}{Q}_{AF,b}$$

**Figure 4:**
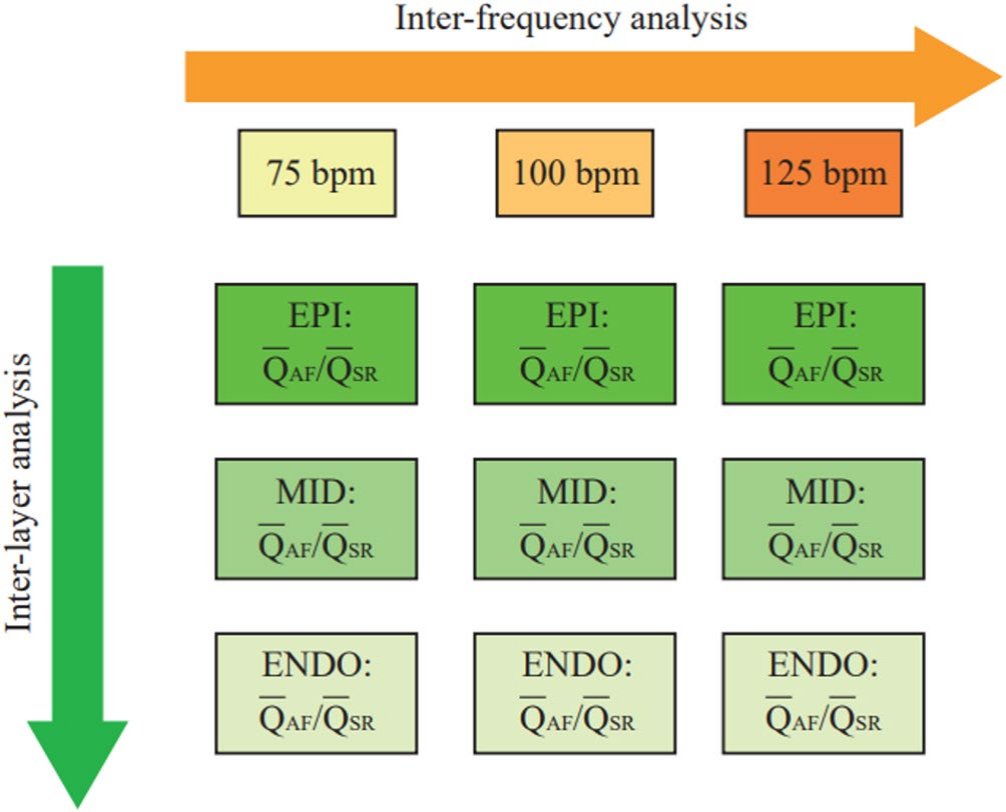
Graphical representation of the two analysis perspectives: (1) inter-layer analysis aims at comparing the ${\overline{Q} }_{AF}$/${\overline{Q} }_{SR}$ ratio, given a specific ventricular rate, across the different myocardial layers (subepicardium, midwall or subendocardium), to assess the differential impact of atrial fibrillation on the perfusion of the different myocardial strata; (2) inter-frequency analysis compares the ${\overline{Q} }_{AF}$/${\overline{Q} }_{SR}$ ratio, given a specific myocardial layer across different simulated ventricular rates, to assess the contribution of ventricular rate to the alteration of the coronary circulation. *EPI* subepicardium, *MID* midwall, *ENDO* subendocardium.

Then, following both an inter-layer and an inter-frequency approach, according to Fig. 4, variations of mean value ${\overline{Q} }_{AF}$/${\overline{Q} }_{SR}$ ratios were evaluated. Significance of all results was proved through statistical tests of hypothesis. ${\overline{Q} }_{SR}$ vs. ${\overline{Q} }_{AF}$ comparison was assessed via Wilcoxon’s test for medians, for all cardiac layers and frequencies, as well as across layers and among frequencies under a given cardiac rhythm (SR or AF). ANOVA tests were also performed on ${Q}_{AF,b}$/${\overline{Q} }_{SR}$ distributions, across layers and among frequencies. T-Student test was performed to evaluate significance of regression trend interpolating ${Q}_{AF,b}$/${\overline{Q} }_{SR}$ distributions across layers, at a given ventricular rate.

Simulations and statistical analyses were carried out through software MATLAB R2020b. A p-value equal to 0.05 was considered statistically significant.

## Results

Table 1 shows the mean ${\overline{Q} }_{AF}$ and ${\overline{Q} }_{SR}$, as well as the ${\overline{Q} }_{AF}$/${\overline{Q} }_{SR}$ ratio, for the six simulations (AF 75 bpm, SR 75 bpm, AF 100 bpm, SR 100 bpm, AF 125 bpm, SR 125 bpm). At each simulated ventricular rate, independently from the myocardial layer, ${\overline{Q} }_{AF}$ was significantly reduced compared to ${\overline{Q} }_{SR}$ (p-values for all ${\overline{Q} }_{AF}$ vs ${\overline{Q} }_{SR}$ comparisons < 0.001).

**Table 1:** Beat-to-beat analysis reporting mean ${\overline{Q} }_{AF}$, ${\overline{Q} }_{SR}$, ${\overline{Q} }_{AF}/{\overline{Q} }_{SR}$ for each simulation, stratified by myocardial layers.

Simulation	${\overline{Q} }_{AF}$ (ml/s)	${\overline{Q} }_{SR}$ (ml/s)	p-value	${\overline{Q} }_{AF}/{\overline{Q} }_{SR}$	AF-SR reduction (%)
**LAD**					
*75 bpm*					
EPI	0.2451 (0.0201)	0.2507 (0.0067)	< 0.001	0.9778	−2.222
MID	0.2749 (0.0235)	0.2832 (0.0074)	< 0.001	0.9707	−2.928
ENDO	0.3138 (0.0348)	0.3255 (0.0097)	< 0.001	0.9641	−3.587
*100 bpm*					
EPI	0.2583 (0.0155)	0.2663 (0.0061)	< 0.001	0.9700	−2.998
MID	0.2891 (0.0215)	0.3007 (0.0075)	< 0.001	0.9614	−3.859
ENDO	0.3294 (0.0391)	0.3455 (0.0114)	< 0.001	0.9533	−4.667
*125 bpm*					
EPI	0.2644 (0.0150)	0.2761 (0.0037)	< 0.001	0.9578	−4.225
MID	0.2954 (0.0188)	0.3117 (0.0057)	< 0.001	0.9478	−5.224
ENDO	0.3360 (0.0418)	0.3581 (0.0113)	< 0.001	0.9383	−6.169
**LCx**					
*75 bpm*					
EPI	0.3364 (0.0275)	0.3440 (0.0091)	< 0.001	0.9779	−2.206
MID	0.3773 (0.0321)	0.3886 (0.0102)	< 0.001	0.9709	−2.913
ENDO	0.4306 (0.0475)	0.4466 (0.0133)	< 0.001	0.9643	−3.573
*100 bpm*					
EPI	0.3544 (0.0212)	0.3652 (0.0083)	< 0.001	0.9702	−2.980
MID	0.3967 (0.0292)	0.4125 (0.0102)	< 0.001	0.9616	−3.841
ENDO	0.4519 (0.0534)	0.4740 (0.0155)	< 0.001	0.9535	−4.650
*125 bpm*					
EPI	0.3627 (0.0144)	0.3786 (0.0022)	< 0.001	0.9580	−4.204
MID	0.4052 (0.0255)	0.4275 (0.0062)	< 0.001	0.9480	−5.203
ENDO	0.4609 (0.0570)	0.4911 (0.0223)	< 0.001	0.9385	−6.149
**RCA**					
*75 bpm*					
EPI	0.0389 (0.0045)	0.0394 (0.0015)	< 0.001	0.9859	−1.406
MID	0.0476 (0.0052)	0.0484 (0.0017)	< 0.001	0.9843	−1.569
ENDO	0.0554 (0.0058)	0.0564 (0.0019)	< 0.001	0.9829	−1.714
*100 bpm*					
EPI	0.0409 (0.0037)	0.0419 (0.0013)	< 0.001	0.9774	−2.259
MID	0.0501 (0.0041)	0.0514 (0.0015)	< 0.001	0.9756	−2.438
ENDO	0.0583 (0.0045)	0.0599 (0.0016)	< 0.001	0.9740	−2.602
*125 bpm*					
EPI	0.0418 (0.0028)	0.0434 (0.0009)	< 0.001	0.9641	−3.589
MID	0.0513 (0.0031)	0.0533 (0.0010)	< 0.001	0.9624	−3.763
ENDO	0.0596 (0.0032)	0.0620 (0.0011)	< 0.001	0.9607	−3.929

*LAD* left anterior descending artery, *EPI* subepicardium, *MID* midwall, *ENDO* subendocardium, *LCx* left circumflex artery, *RCA* right coronary artery.

**Figure 5:**
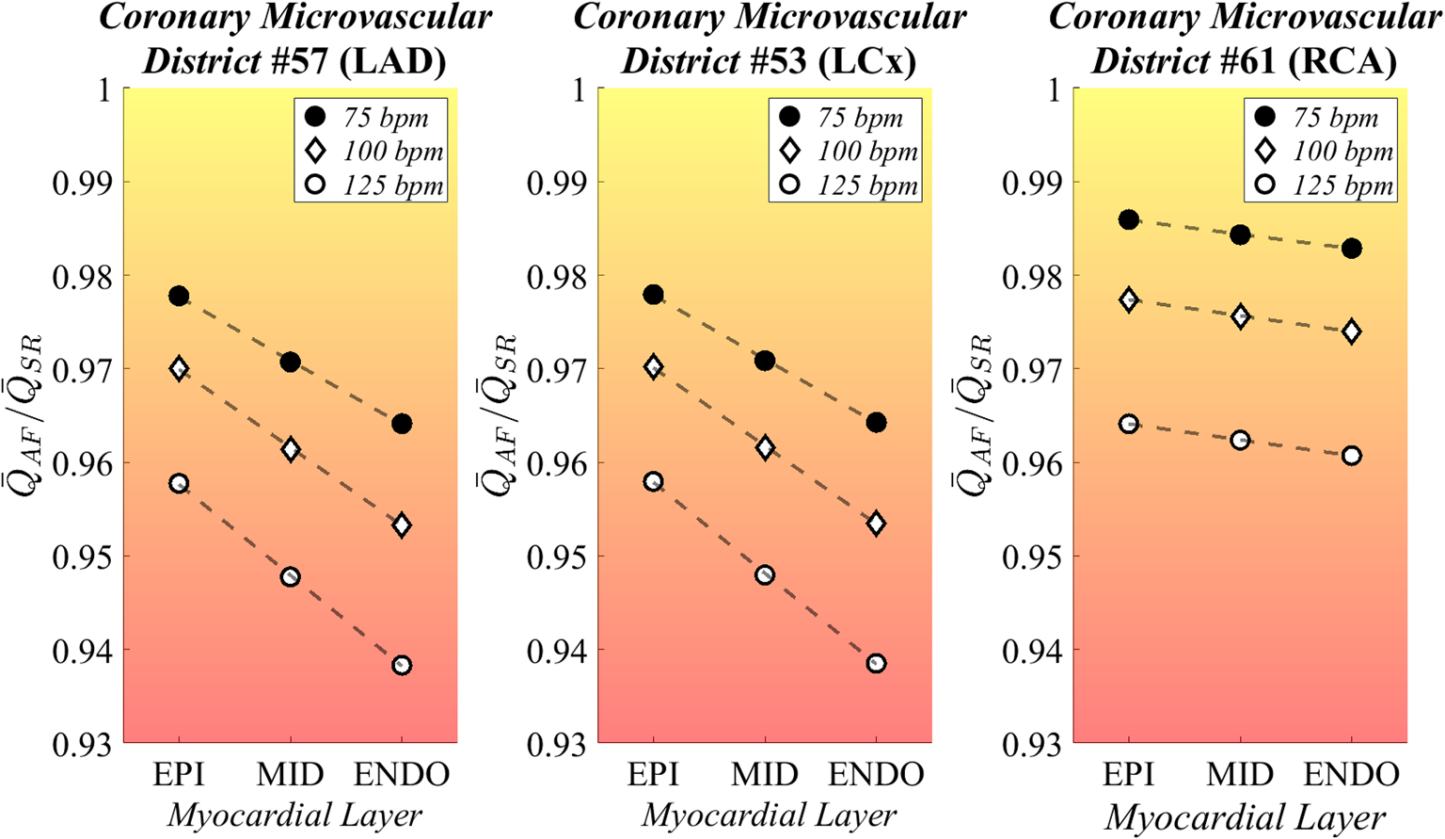
Dot plot reporting ${\overline{Q} }_{AF}$/${\overline{Q} }_{SR}$ at each simulated frequency across the myocardial layers, in three exemplificative coronary microvascular districts. Rate-specific regression lines (dashed lines) are also reported. *LAD* left anterior descending artery, *EPI* subepicardium, *MID* midwall, *ENDO* subendocardium, *LCx* left circumflex artery, *RCA* right coronary artery.

The inter-layer and inter-frequency ANOVA test results with the corresponding p-values are reported in Table 2. Figure 5 shows, for each coronary district, the ${\overline{Q} }_{AF}$/${\overline{Q} }_{SR}$ ratio across cardiac layers for the investigated ventricular rates. Inter-layer analysis showed that, for each simulated ventricular rate, ${\overline{Q} }_{AF}$/${\overline{Q} }_{SR}$ progressively decreased from the epicardial to the endocardial layer in the distal left coronary artery districts (p-values < 0.001 for both LAD and LCx), while this was not the case for the distal RCA district (p-value 0.669, 0.409, 0.186 for 75 bpm, 100 bpm and 125 bpm simulations, respectively). Focusing on inter-frequency analysis, ${\overline{Q} }_{AF}$/${\overline{Q} }_{SR}$ progressively worsened as the ventricular rates increased. In fact, in all the investigated distal coronary artery districts (LAD, LCx and RCA), we found a significant decrease in ${\overline{Q} }_{AF}$/${\overline{Q} }_{SR}$ evaluated at cardiac layer when comparing the different ventricular rates (p-values < 0.001 for all layer-specific comparisons).

**Table 2:** Inter-layer (comparison of ${Q}_{AF,b}$/${\overline{Q} }_{SR}$ across the different myocardial layers given a specific ventricular rate) and inter-frequency (comparison of ${Q}_{AF,b}$/${\overline{Q} }_{SR}$ across the different simulated ventricular rates given a specific myocardial layer) ANOVA test results.

Inter-layer analysis (EPI vs MID vs ENDO)	p-value
**LAD**	
75 bpm	< 0.001
100 bpm	< 0.001
125 bpm	< 0.001
**LCx**	
75 bpm	< 0.001
100 bpm	< 0.001
125 bpm	< 0.001
**RCA**	
75 bpm	0.669
100 bpm	0.409
125 bpm	0.186
**Inter-frequency analysis (75 bpm vs 100 bpm vs 125 bpm)**	
**LAD**	
EPI	< 0.001
MID	< 0.001
ENDO	< 0.001
**LCx**	
EPI	< 0.001
MID	< 0.001
ENDO	< 0.001
**RCA**	
EPI	< 0.001
MID	< 0.001
ENDO	< 0.001

*EPI* subepicardium, *MID* midwall, *ENDO* subendocardium, *LAD* left anterior descending artery, *LCx* left circumflex artery, *RCA* right coronary artery.

Finally, inter-frequency analysis assessing potential differences in the slopes of ${Q}_{AF,b}$/${\overline{Q} }_{SR}$ across cardiac layers did not report significant differences, albeit a trend towards significance was evident in comparing the slopes at 75 and 125 bpm in the left microvascular coronary artery districts (p-values 0.129 for both LAD and LCx). Supplementary Table 6 reports the slopes of the rate-specific ${Q}_{AF,b}$/${\overline{Q} }_{SR}$ regression lines at each microvascular district (p-values for pairwise comparisons).

## Discussion

The main findings of the present computational analysis, based on a 1D-0D multiscale model of the entire human cardiovascular system enriched by a detailed mathematical modeling of the coronary arteries and their downstream microcirculatory districts, are the following:

- AF exerts direct hemodynamics consequences on the coronary microcirculation which might partly explain angina-like symptoms, particularly at high ventricular rates, if superimposed on a susceptible substrate (i.e.*,* hypertensive microvascular coronary disease, AF-related endothelial dysfunction);
- Considering a constant mean ventricular rate, AF, compared to SR, decreased the mean blood flow at each myocardial layer (particularly in the subendocardial layer);
- Given a specific myocardial layer, higher ventricular rates during AF relate to a more pronounced reduction in microvascular blood flow, if compared to the corresponding SR simulation.

It has been extensively described that AF patients may suffer from ischemic chest pain, with associated electrocardiographic changes (ST depression), even in case of normal epicardial coronary arteries^4–6^. In particular, the magnitude of ischemic electrocardiographic alterations (ST depression) during ongoing AF (specially at high ventricular rates) do not seem to be predictive of obstructive epicardial coronary artery disease, differently from SR where electrocardiographic changes are clear predictors of obstructive epicardial coronary disease^28^. This apparently ambiguous finding is partly explained by AF-induced coronary microvascular dysfunction, previously documented in vivo and accounting for a blunted coronary flow reserve in AF patients.

However, another possible mechanism may be the direct hemodynamic influence exerted by the classically irregularly irregular AF rhythm on the coronary circle. The coronary circulation is peculiar, blood flow is prominent during diastole, due to the complex interplay between the forcing pressure (aortic pressure) and the extravascular forces (myocytes contraction and left ventricular end-diastolic pressure) that compress the microvasculature of the different myocardial layers, in particular the subendocardium^29^. Surprisingly, this mechanism has not yet been thoroughly investigated in this context, albeit clinical data in human clearly demonstrated that the irregular beating of AF produces detrimental hemodynamic effects in terms of decreased cardiac output and increased biventricular filling pressure^30^. Our group was the first to demonstrate that AF can exert direct epicardial coronary flow impairment and oxygen supply–demand ratio unbalancing based on a multiscale computational model^18^. For this reason, we aimed to deepen the comprehension of the direct hemodynamic effect of AF on the coronary circulation, by designing a novel in silico approach implementing a complex multiscale 1D-0D computational model enriched by detailed mathematical description of the coronary microcirculatory districts. The present, therefore, is the first study suggesting detrimental consequences that an irregularly irregular rhythm per se exerts on microvascular coronary blood flow. On top of AF-related endothelial dysfunction, the altered rhythm may itself directly decrease myocardial blood flow, particularly at the subendocardial level. This reduction in myocardial perfusion appears to be dependent on ventricular rate, with greater reduction observed at higher rates during AF, another potential element favoring a stricter rate control target in patients with permanent AF^10,31^. Our findings are in line with previous evidence on animals: Saito et al.^32^ demonstrated in anesthetized open-chest dogs that mechanically-induced AF diminishes coronary flow reserve, particularly in subendocardial layers (subendocardial blood flow was reduced by 22%, while subepicardial blood flow by 9% only). Kochiadakis et al.^33^ similarly demonstrated in humans a reduced coronary flow reserve in experimentally induced AF compared to right atrial pacing at a similar heart rate, even if they did not assess potential differences in blood flow distribution across myocardial wall.

Moreover, the computational framework here adopted is not merely able to describe AF-induced direct hemodynamic effects, but also unique details regarding the possible mechanisms behind these phenomena. In fact, as reported in Supplementary Table 7, an in-depth analysis of two key factors of coronary hemodynamics, such as the driving pressure (aortic pressure) and the most relevant extravascular force (left ventricular end-diastolic pressure) suggests that: (1) the reduced coronary flow during AF, compared to SR, at higher ventricular rates correlates to a drop in mean aortic pressure (and consequent reduced cardiac output and coronary circulation driving pressure); (2) the greater reduction in subendocardial perfusion, relative to the other cardiac layers, during AF in the left-sided coronary arteries (LAD and LCx) correlates to a significantly increased left ventricular end diastolic pressure (further worsened at faster ventricular rates; not relevant in the right ventricle given the lower absolute values of the endoventricular pressures).

### Limitations

The present computational work presents the following limitations. The coronary microvascular model includes an autoregulation mechanism, but does not directly account for metabolic regulations, as well as for AF-induced endothelial dysfunction. In addition, the presence of other comorbidities and the potential hemodynamic effects of rate control drugs is not taken into account. However, considering that the focus of our analysis was to assess the pure hemodynamic (i.e. not endothelial dysfunction-mediated) effect of AF on coronary circulation, the simplified but powerful computational framework, as well as the use of standardized conditions regardless of any other baseline clinical feature that could potentially alter mechanical properties of both cardiac vessels and cardiac muscle, allows to explore the standalone impact that the irregular AF beating exerts on the coronary circle.

## Conclusions

Based on a 1D-0D multiscale model of the entire human cardiovascular system, AF exerts direct hemodynamics consequences on the coronary microcirculation. Microvascular coronary flow is, in fact, reduced during AF compared to SR, particularly at higher ventricular rates; in addition, for left coronary arteries (LAD and LCx), a significant gradient in subendocardial-subepicardial perfusion was demonstrated, with left subendocardial layers suffering the most evident blood flow decrease during the arrhythmia.

## Supplementary Information

Supplementary Information.

## Data Availability

Data available on request.
