# Supplementary material for: A computational analysis of atrial fibrillation effects on coronary perfusion across the different myocardial layers
Source: Sci Rep. 2022 Jan 17;12:841. doi: 10.1038/s41598-022-04897-6 (PMC8763927; doi:10.1038/s41598-022-04897-6)

**A computational analysis of atrial fibrillation effects**

**on coronary perfusion across the different myocardial layers**

*Supplementary Information*

Andrea Saglietto^1^, Matteo Fois^2^, Luca Ridolfi^3^, Gaetano Maria De Ferrari^1^, Matteo Anselmino^1*#^, Stefania Scarsoglio^2#^

^1^ Division of Cardiology, “Città della Salute e della Scienza di Torino” Hospital, Department of Medical Sciences, University of Turin, Turin, Italy;

^2^ Department of Mechanical and Aerospace Engineering, Politecnico di Torino, Turin, Italy;

^3^ Department of Environmental, Land and Infrastructure Engineering, Politecnico di Torino, Turin, Italy.

*Corresponding author:

Matteo Anselmino; email address: matteo.anselmino@unito.it; address: C.so Dogliotti 14, Torino; telephone number: (39)-0116709598

# These two Authors contributed equally (co-last position)

Conflict of interests: none

Data availability statement: data available on request

**Mathematical model**

In this section, model mathematical details are synthetically presented. Equations (S1-S2) in the red box describe blood motion (mass and momentum conservation) within the 1D arterial network, where variables $A(x,t)$*,* $Q(x,t)$ are the vessel cross section area and the blood flow rate, respectively ($x$ and $t$ are the axial and time coordinates). Parameters $\beta$ and $N_{4}$ are the Coriolis and viscous coefficient, respectively, depending on the assumed velocity profile and blood properties. Blood pressure $p(x,t)$ is included through equation (S3), where coefficients $B_{i}$ (*i*=1…5) are calculated via equations (S4-S9) depending on vessels mechanical properties (accounting for their visco-elastic behavior). Geometric properties and parameters of the 1D arterial tree are enclosed in Supplementary Table 1 (*h_w0_* and *r_0_* are initial arterial wall thickness lumen radius, respectively, whereas *a_2_*=13.3 m^1.3^/s, *b_2_*=0.3, *a_3_*=1914 N^2/3^/m^4/3^, *a_5_*=-45348 N/m^2^ and *K_visc_* are constant parameters).

Equations (S10-S11) in the red box represent mass and total pressure conservation imposed at arterial bifurcations ($\rho$ is blood density, subscript 0 denotes the parent vessel, subscript 1,2 refer to the daughter vessels). A third daughter vessel is added for the case of the coronary bifurcation. Equation (S12) allows for computation of the characteristic impedances (attached to each 0D arteriolar and coronary microvasculature inlet branch, with $A_{out}$ the outlet cross section area of the adjacent 1D artery), and time-dependent pressure wave velocity $PWV$ is obtained through equation (S13).

Equations (S14-S17) in the blue box regulate 0D compartments mass and momentum balance. In equations (S14-S17), subscript *j* identifies the *j*-th 0D compartment, so that *j*-1 and *j*+1 refer to the previous and following compartments, respectively. $V_{j}$ is the *j*-th compartmental total blood volume, $Q_{j}$ is the blood flow rate, whereas $R_{j}$ and $L_{j}$ are compartmental lumped resistance and inertance, respectively. Equation (S17) represents the constitutive equation for the compartmental blood pressure $p_{j}$ ($V_{j,un}$ is the compartmental unstressed blood volume, $C_{j}$ is compartmental compliance, $p_{j}-p_{j}^{ext}$ is transmural pressure, with $p_{j}^{ext}$ extravascular pressure, which is null for all compartments but the coronary miscovasculature). All 0D lumped parameter settings are reported in Supplementary Table 2.

Equations (S18-S21) in the yellow box describe the cardiac chambers functioning. $V_{ch}$ and $V_{ch,un}$ are the chambers total and unstressed volume, while $E_{ch}$ is the *ch*-th chamber’s elastance function. In equation (S19), $E_{ch,A}$*,* $E_{ch,B}$ and $e_{ch}$, are the *ch*-th chamber’s elastance amplitude, minimum value, and normalized time-varying function, respectively. This latter assumes a different formulation for atria and for ventricles, *i.e.* $e_{a}$or $e_{v}$, expressed by equations (S20-S21), where $t_{ar}$, $t_{ac}$, $T_{ar}$, $T_{ac}$, $T_{vr}$, $T_{vc}$ are atria (*a*)/ventricles (*v*) relaxation (*r*)/contraction (*c*) starting time (*t*)/period (*T*), respectively. $RR$ is the heart-beat duration. Parameters involved in the 0D cardiac model are reported in Supplementary Table 3.

Equations (S22-S23) regulate cardiac valve’s blood flow rate $Q_{va}$ and opening angle $\theta_{va}$, where $R_{va}$, $B_{va}$ and $L_{va}$ are the lumped viscous resistance, Bernoulli’s coefficient and inertance associated with the valve, respectively. Coefficients $k_{p,va}$, $k_{q,va}$, $k_{f,va}$ and $k_{v,va}$ represent pressure, dynamics, viscous and downstream vortexes actions onto each valve’s leaflets. Valves parameter settings are also included in Supplementary Table 3.

Short-term baroreflex control is modelled through equations (S24-S27) in the green box, where the time evolution of the *m*-th efferent organ $y_{m}$ depends upon the saturation parameters of the sympathetic ($\alpha_{m}$) and parasympathetic ($\beta_{m}$) response for the corresponding *m*-th efferent organ, as well as on its time delay ($\tau_{m}$) and the complete denervation parameter ($\gamma_{m}$). We recall that the heart rate is not involved in the baroreflex control, since it is externally imposed as the primary input of the model (simulations were performed by specifying the mean pacing frequency under sinus rhythm and atrial fibrillation). $\bar{p}_{acs}$ and $\bar{p}_{acs,tg}$ are the current and target aortic-carotid sinus pressure (calculated as the average pressure – over the cardia cycle -of the aortic arch, right and left carotid sinus pressures, respectively $p_{AA}$, $p_{cs,R}$, $p_{cs,L}$) involved in the baroreflex control, respectively, and $\nu$ is the steepness of response of the sympathetic ($n_{s}$) and parasympathetic ($n_{p}$) activity. Baroreflex control parameter settings are reported in Supplementary Table 4.

Equations (S28-S42) in the purple box describe all lumped parameters involved in the 0D model of the coronary miscrovasculature. For the *k*-th 0D coronary district, arterial ($C_{1,jj,k}$) and venous ($C_{3,jj,k}$) compliances of the *jj*-th layer (*jj*=1 subepicardium, *jj*=2 midwall, *jj*=3 subendocardium) are calculated through equations (S28-S29). $C_{1,T}$ and $C_{3,T}$ are the total arterial and venous coronary compliance, $r_{k}$ is the outer radius of the upstream 1D coronary vessel, $W_{G}$ is the weight of the myocardial region $G$ (left ventricle, septum or right ventricle) and $\gamma_{jj}$ is a vector allowing for the compliance repartition over the three layers. Similarly, initial (subscript 0) total blood volumes ($V_{0,1,jj,k}$ and $V_{0,3,jj,k}$) of each 0D coronary district are determined through equations (S30-S31). Equations (S32-S33) describe the computation of inlet arterial ($C_{a,k}$) and outlet venous ($C_{v,k}$) compliances of the *k*-th 0D coronary district, respectively. Lumped coronary initial (subscript 0) resistances of the *k*-th 0D district are obtained via equations (S34-S37), where $R_{0,s,jj,k}$ is the summation of the arterial ($R_{0,1,jj,k}$), intermediate ($R_{0,2,jj,k}$) and venous ($R_{0,3,jj,k}$) resistances pertaining to the *jj*-th myocardial layer, and $R_{0,jj,T,G}$ is the total resistance of the *jj*-th layer associated with the myocardial region $G$. Outlet venous impedance $Z_{v,k}$ of the *k*-th 0D coronary district is computed through equation (S31), depending upon inlet arterial impedance $Z_{a,k}$, obtained via equation (S12). Non-linear behavior of time-varying coronary resistances ($R_{1,jj,k}$, $R_{2,jj,k}$ and $R_{3,jj,k}$) is described through equations (S39-S41), based on the corresponding time dependent total blood volume ($V_{1,jj,k}$ and $V_{3,jj,k}$). Coronary microvasculature extravascular pressure - *i.e.* intramyocardial pressure $p_{jj}^{im}$ - acting onto the *jj*-th myocardial layer, is computed through equation (S42), accounting for the effect of cavity-induced extracellular pressure ($CEP$, depending upon the adjacent ventricular pressure $p_{v}$ (right, left or both ventricles) throughout the repartition vector over the three layers $\gamma_{CEP,jj}$) and of shortening-induced intracellular pressure ($SIP$, depending on the corresponding ventricular elastance function $E_{v}$, through a constant parameter $\varphi_{SIP}$). All 0D coronary microvasculature parameters and tuning values are enclosed in Supplementary Table 5.

***1D arterial Tree***

$$\left( S1 \right) \frac{\text{∂A}}{\text{∂t}}\text{+}\frac{\text{∂Q}}{\text{∂x}}\text{=0}$$

$$\left( S2 \right) \frac{\text{∂Q}}{\text{∂t}}\text{+}\frac{\text{∂}}{\text{∂x}}\left( \text{β}\frac{Q^{2}}{A} \right)=-\frac{A}{\rho}\frac{\text{∂}p}{\text{∂x}}+N_{4}\frac{Q}{A}$$

$$\left( S3 \right) p=B_{1}+B_{2}A+B_{3}A^{2}+B_{4}A^{3}-B_{5}\frac{1}{\sqrt{A}}\frac{\text{∂Q}}{\text{∂x}}$$

$$\left( S4 \right) B_{1}=-\frac{1}{a_{3}}\left( a_{5}^{3}+\mathrm{PWV}_{0}^{6}\rho^{3}+3\mathrm{PWV}_{0}^{4}\rho^{2}a_{5}+3a_{5}^{2}\mathrm{PWV}_{0}^{2}\rho\right)$$

$$\left( S5 \right) B_{2}=\frac{3\rho\mathrm{PWV}_{0}^{2}}{A_{0}a_{3}^{3}}\left( a_{5}^{2}+\mathrm{PWV}_{0}^{4}\rho^{2}+2a_{5}\mathrm{PWV}_{0}^{2}\rho\right)$$

$$\left( S6 \right) B_{3}=-\frac{3\rho^{2}\mathrm{PWV}_{0}^{4}}{A_{0}^{2}a_{3}^{3}}\left( a_{5}+\mathrm{PWV}_{0}^{2}\rho\right)$$

$$\left( S7 \right) B_{4}=\left( \frac{\rho\mathrm{PWV}_{0}^{2}}{a_{3}A_{0}} \right)^{3}$$

$$\left( S8 \right) B_{5}=\frac{K_{visc}h_{w0}}{r_{0}\sqrt{A_{0}}}$$

$$\left( S9 \right) \mathrm{PWV}_{0}=\frac{a_{2}}{d_{0}^{b_{2}}}$$

$$\left( S10 \right) Q_{0}=Q_{1}+Q_{2}$$

$$\left( S11 \right) p_{0}+\frac{1}{2}\rho\left( \frac{Q_{0}}{A_{0}} \right)^{2}=p_{1}+\frac{1}{2}\rho\left( \frac{Q_{1}}{A_{1}} \right)^{2}=p_{2}+\frac{1}{2}\rho\left( \frac{Q_{2}}{A_{2}} \right)^{2}$$

$$\left( S12 \right) Z_{c}=\frac{\rho\mathrm{PWV}_{0}}{A_{\mathrm{out}}}$$

$\left( S13 \right) PWV=\sqrt{\frac{A}{\rho}\left( B_{2}+2B_{3}A+3B_{4}A^{2} \right)+\frac{Q^{2}}{A^{2}}\beta\left( \beta-1 \right)}$

***0D Microcirculation, Venous Return and Pulmonary***

$$\left( S14 \right) \frac{dV_{j}}{\mathrm{dt}}=Q_{j-1}-Q_{j}$$

$$\left( S15 \right) \frac{dQ_{j}}{\mathrm{dt}}=\frac{p_{j}-R_{j}Q_{j}-p_{j+1}}{L_{j}}$$

$$\left( S16 \right) \frac{dp_{j}}{\mathrm{dt}}=\frac{Q_{j-1}-Q_{j}}{C_{j}}$$

$$\left( S17 \right) V_{j}=V_{j,un}+\left( p_{j}-p_{j}^{\mathrm{ext}} \right)C_{j}$$

***0D Heart and Valves***

$$\left( S18 \right) p_{\mathrm{ch}}=E_{\mathrm{ch}}\left( V_{\mathrm{ch}}-V_{ch,un} \right)$$

$$\left( S19 \right) E_{\mathrm{ch}}=E_{ch,A}e_{\mathrm{ch}}+E_{ch,B}$$

$$\left( S20 \right) e_{a}=\left\{ \begin{aligned} \frac{1}{2}\left[ 1+\cos\left( \pi\frac{t+RR-t_{\mathrm{ar}}}{T_{\mathrm{ar}}} \right) \right];0\leq t\leq t_{\mathrm{ar}}+T_{\mathrm{ar}}-RR \\ 0 ; t_{\mathrm{ar}}+T_{\mathrm{ar}}-RR\leq t\leq t_{\mathrm{ac}} \\ \frac{1}{2}\left[ 1-\cos\left( \pi\frac{t-t_{\mathrm{ac}}}{T_{\mathrm{ac}}} \right) \right] ; t_{\mathrm{ac}}\leq t\leq t_{\mathrm{ac}}+T_{\mathrm{ac}} \\ \frac{1}{2}\left[ 1+\cos\left( \pi\frac{t-t_{\mathrm{ar}}}{T_{\mathrm{ar}}} \right) \right]; t_{\mathrm{ac}}+T_{\mathrm{ac}}\leq t\leq RR \end{aligned} \right.$$

$$\left( S21 \right) e_{v}=\left\{ \begin{aligned} \frac{1}{2}\left[ 1-\cos\left( \pi\frac{t}{T_{\mathrm{vc}}} \right) \right]; 0\leq t\leq T_{\mathrm{vc}} \\ \frac{1}{2}\left[ 1+\cos\left( \pi\frac{t-T_{\mathrm{vc}}}{T_{\mathrm{vr}}} \right) \right]; T_{\mathrm{vc}}\leq t\leq T_{\mathrm{vc}}+T_{\mathrm{vr}} \\ 0 ; T_{\mathrm{vc}}+T_{\mathrm{vr}}\leq t\leq RR \end{aligned} \right.$$

$$\left( S22 \right) L_{\mathrm{va}}\frac{dQ_{\mathrm{va}}}{\mathrm{dt}}+R_{\mathrm{va}}Q_{\mathrm{va}}+B_{\mathrm{va}}\left| Q_{\mathrm{va}} \right|Q_{\mathrm{va}}=\frac{\left( 1-\cos\theta_{\mathrm{va}} \right)^{4}}{\left( 1-\cos\theta_{va,max} \right)^{4}}\Delta p_{\mathrm{va}}$$

$$\left( S23 \right) L_{\mathrm{va}}\frac{d^{2}\theta_{\mathrm{va}}}{dt^{2}}=k_{p,va}\Delta p_{\mathrm{va}}+k_{q,va}Q_{\mathrm{va}}\cos\theta_{\mathrm{va}}-k_{f,va}\frac{d\theta_{\mathrm{va}}}{\mathrm{dt}}-k_{v,va}\sin2\theta_{\mathrm{va}}$$

***Short-Term Baroreflex Control***

$$\left( S24 \right) \frac{dy_{m}}{\mathrm{dt}}=\frac{1}{\tau_{m}}\left( -y_{m}+\alpha_{m}n_{s}-\beta_{m}n_{p}+\gamma_{m} \right)$$

$$\left( S25 \right) n_{s}=\frac{1}{1+\left( \frac{\bar{p}_{\mathrm{acs}}}{\bar{p}_{acs,tg}} \right)^{\nu}}$$

$$\left( S26 \right) n_{p}=\frac{1}{1+\left( \frac{\bar{p}_{\mathrm{acs}}}{\bar{p}_{acs,tg}} \right)^{-\nu}}$$

$$\left( S27 \right) \bar{p}_{\mathrm{acs}}=\frac{1}{3RR}\int_{\mathrm{RR}} \left( p_{\mathrm{AA}}\left( t \right)+p_{cs,R}\left( t \right)+p_{cs,L}(t) \right)\mathrm{dt}$$

***0D Coronary Microvasculature***

$$\left( S28 \right) C_{1,jj,k}=C_{1,T}\frac{W_{G}}{\sum_{k\in G} r_{k}^{3}}\gamma_{\mathrm{jj}}$$

$$\left( S29 \right) C_{3,jj,k}=C_{3,T}\frac{W_{G}}{\sum_{k\in G} r_{k}^{3}}\gamma_{\mathrm{jj}}$$

$$\left( S30 \right) V_{0,1,jj,k}=V_{0,1,T}\frac{W_{G}}{\sum_{k\in G} r_{k}^{3}}\gamma_{\mathrm{jj}}$$

$$\left( S31 \right) V_{0,3,jj,k}=V_{0,3,T}\frac{W_{G}}{\sum_{k\in G} r_{k}^{3}}\gamma_{\mathrm{jj}}$$

$$\left( S32 \right) C_{a,k}=\frac{1}{10}C_{1,T}\frac{W_{G}}{\sum_{k\in G} r_{k}^{3}}$$

$$\left( S33 \right) C_{v,k}=\frac{1}{10}C_{3,T}\frac{W_{G}}{\sum_{k\in G} r_{k}^{3}}$$

$$\left( S34 \right) R_{0,s,jj,k}=\frac{R_{0,jj,T,G}}{r_{k}^{3}\sum_{k\in G} r_{k}^{-3}}$$

$$\left( S35 \right) R_{0,2,jj,k}=\frac{R_{0,s,jj,k}}{2.7}$$

$$\left( S36 \right) R_{0,1,jj,k}=1.1R_{0,2,jj,k}$$

$$\left( S37 \right) R_{0,3,jj,k}=0.9R_{0,2,jj,k}$$

$$\left( S38 \right) Z_{v,k}=\frac{Z_{a,k}}{{1.4}^{2}}$$

$$\left( S39 \right) R_{1,jj,k}=R_{0,1,jj,k}\left( \frac{V_{0,1,jj,k}}{V_{1,jj,k}} \right)^{2}$$

$$\left( S40 \right) R_{3,jj,k}=R_{0,3,jj,k}\left( \frac{V_{0,3,jj,k}}{V_{3,jj,k}} \right)^{2}$$

$$\left( S41 \right) R_{2,jj,k}=R_{0,2,jj,k}\left[ 0.75\left( \frac{V_{0,1,jj,k}}{V_{1,jj,k}} \right)^{2}+0.25\left( \frac{V_{0,3,jj,k}}{V_{3,jj,k}} \right)^{2} \right]$$

$$\left( S42 \right) p_{\mathrm{jj}}^{\mathrm{im}}=CEP+SIP=p_{v}\gamma_{CEP,jj}+\varphi_{\mathrm{SIP}}E_{v}$$

**Supplementary Table 1.** 1D arterial tree and coronary circulation vessels’ nomenclature and geometry (*d_in,0_*, *d_out,0_*, and *l* are arterial vessels initial inlet and outlet diameter, and length, respectively).

| **#id** | **name** | **d_in,0_ [mm]** | **d_out,0_ [mm]** | **l [mm]** | **h_w0_ [mm]** | **K_visc_ [mmHg s]** |
| --- | --- | --- | --- | --- | --- | --- |
| 1 | Ascending Aorta I | 29.4 | 29.3 | 10 | 1.6 | 33 |
| 2 | Aortic Arch I | 24.1 | 24 | 20 | 1.3 | 33 |
| 3 | Brachiocephalic Artery | 19.4 | 18 | 34 | 0.9 | 33 |
| 4 | R Subclavian Artery I | 12.9 | 9 | 34 | 0.7 | 33 |
| 5 | R Common Carotid Artery | 15.1 | 7 | 94 | 0.6 | 33 |
| 6 | R Vertebral Artery | 4.1 | 2.8 | 149 | 0.5 | 67 |
| 7 | R Subclavian II, Axillary & Brachial Artery | 8.9 | 4.7 | 422 | 0.7 | 33 |
| 8 | R Radial Artery | 3.7 | 3.1 | 235 | 0.4 | 67 |
| 9 | R Ulnar Artery I | 3.7 | 3.4 | 67 | 0.5 | 67 |
| 10 | R Interosseous Artery | 2.1 | 1.8 | 79 | 0.3 | 134 |
| 11 | R Ulnar Artery II | 3.2 | 2.8 | 171 | 0.5 | 67 |
| 12 | R Internal Carotid Artery | 5.7 | 4.3 | 178 | 0.4 | 67 |
| 13 | R External Carotid Artery | 5 | 4.5 | 41 | 0.4 | 67 |
| 14 | Aortic Arch II | 22 | 20.8 | 39 | 1.3 | 33 |
| 15 | L Common Carotid Artery | 12.4 | 6 | 139 | 0.6 | 33 |
| 16 | L Internal Carotid Artery | 5 | 4.1 | 178 | 0.4 | 67 |
| 17 | L External Carotid Artery | 4.5 | 4.1 | 41 | 0.4 | 67 |
| 18 | Thoracic Aorta I | 20 | 18.9 | 52 | 1.2 | 33 |
| 19 | L Subclavian Artery I | 11 | 8.5 | 34 | 0.7 | 33 |
| 20 | L Vertebral Artery | 3.8 | 2.8 | 148 | 0.5 | 67 |
| 21 | L Subclavian II, Axillary & Brachial Artery | 8.4 | 4.7 | 422 | 0.7 | 33 |
| 22 | L Radial Artery | 3.3 | 2.8 | 235 | 0.4 | 67 |
| 23 | L Ulnar Artery I | 4 | 4 | 67 | 0.5 | 67 |
| 24 | L Interosseous Artery | 1.8 | 1.8 | 79 | 0.3 | 134 |
| 25 | L Ulnar Artery II | 4.1 | 3.7 | 171 | 0.5 | 67 |
| 26 | Intercostal Arteries | 12.6 | 9.5 | 80 | 1.2 | 67 |
| 27 | Thoracic Aorta II | 16.5 | 12.9 | 104 | 1.2 | 33 |
| 28 | Abdominal Aorta I | 12.2 | 12.2 | 53 | 1.1 | 33 |
| 29 | Celiac Artery I | 7.8 | 6.9 | 20 | 0.6 | 33 |
| 30 | Celiac Artery II | 5.2 | 4.9 | 25 | 0.6 | 33 |
| 31 | Hepatic Artery | 5.4 | 4.4 | 66 | 0.5 | 33 |
| 32 | Gastric Artery | 3.2 | 3 | 71 | 0.5 | 33 |
| 33 | Splenic Artery | 4.2 | 3.9 | 63 | 0.5 | 33 |
| 34 | Superior Mesenteric Artery | 7.9 | 7.1 | 59 | 0.7 | 33 |
| 35 | Abdominal Aorta II | 11.5 | 11.3 | 20 | 0.8 | 33 |
| 36 | R Renal Artery | 4.9 | 4.9 | 32 | 0.5 | 33 |
| 37 | Abdominal Aorta III | 11.2 | 11.2 | 20 | 0.8 | 33 |
| 38 | L Renal Artery | 4.9 | 5.2 | 32 | 0.5 | 33 |
| 39 | Abdominal Aorta IV | 11 | 11 | 106 | 0.8 | 33 |
| 40 | Inferior Mesenteric Artery | 4.7 | 3.2 | 50 | 0.4 | 33 |
| 41 | Abdominal Aorta V | 10.8 | 10.4 | 20 | 0.8 | 33 |
| 42 | Common Iliac Artery | 7.9 | 7 | 59 | 0.8 | 33 |
| 43 | Inner Iliac Artery | 4 | 4 | 50 | 0.4 | 134 |
| 44 | External Iliac Artery | 6.4 | 6.1 | 144 | 0.6 | 33 |
| 45 | Deep Femoral Artery | 4 | 3.7 | 126 | 0.5 | 134 |
| 46 | Femoral Artery | 5.2 | 3.8 | 443 | 0.5 | 67 |
| 47 | Anterior Tibial Artery | 2.6 | 2.3 | 343 | 0.4 | 134 |
| 48 | Posterior Tibial Artery | 3.1 | 2.8 | 321 | 0.5 | 134 |
| 49 | Left Main Coronary Artery | 4.5 | 4.5 | 12 | 0.4 | 134 |
| 50 | Left Anterior Descending Coronary Artery I | 3.7 | 3.7 | 18 | 0.4 | 134 |
| 51 | Circumﬂex Coronary Artery I | 3.6 | 3.6 | 32 | 0.4 | 134 |
| 52 | Marginal Coronary Artery | 2.5 | 2.5 | 73 | 0.3 | 134 |
| 53 | Circumﬂex Coronary Artery II | 3 | 3 | 43 | 0.3 | 134 |
| 54 | Diagonal Coronary Artery | 2.4 | 2.4 | 65 | 0.2 | 134 |
| 55 | Left Anterior Descending Coronary Artery II | 3.3 | 3.3 | 22 | 0.3 | 134 |
| 56 | Septal Coronary Artery II | 2.4 | 2.4 | 45 | 0.2 | 134 |
| 57 | Left Anterior Descending Coronary Artery III | 2.7 | 2.7 | 100 | 0.3 | 134 |
| 58 | Right Coronary Artery I | 4.1 | 4.1 | 50 | 0.4 | 134 |
| 59 | Acute Marginal Coronary Artery I | 2.5 | 2.5 | 34 | 0.3 | 134 |
| 60 | Right Coronary Artery II | 3.6 | 3.6 | 48 | 0.4 | 134 |
| 61 | Acute Marginal Coronary Artery II | 2.1 | 2.1 | 23 | 0.2 | 134 |
| 62 | Right Coronary Artery III | 3.2 | 3.2 | 89 | 0.3 | 134 |
| 63 | Ascending Aorta II | 29.3 | 28.8 | 30 | 1.6 | 33 |

**Supplementary Table 2.** 0D systemic and pulmonary circulation parameter settings (LB and UB refer to lower body and upper body compartments, respectively).

| **0D Arterioles** | | | | | | |
| --- | --- | --- | --- | --- | --- | --- |
| **#id** | **Name** | **R [mmHg s/ml]** | **C [ml/mmHg]** | **L [mmHg s/ml]** | **V_un_ [ml]** | **V [ml]** |
| 6 | R Vertebral | 34.94 | 0.013 | 0.019 | 4.2 | 4.72 |
| 8 | R Radial | 22.99 | 0.014 | 0.018 | 3.6 | 4.16 |
| 10 | R Interosseous | 531.50 | 0.0009 | 0.07 | 3.6 | 3.64 |
| 11 | R Ulnar II | 26.58 | 0.014 | 0.018 | 3.6 | 4.16 |
| 12 | R Internal Carotid | 31.86 | 0.015 | 0.017 | 4.2 | 4.80 |
| 13 | R External Carotid | 29.50 | 0.015 | 0.017 | 4.2 | 4.80 |
| 16 | L Internal Carotid | 31.86 | 0.015 | 0.017 | 4.2 | 4.80 |
| 17 | L External Carotid | 29.50 | 0.015 | 0.017 | 4.2 | 4.80 |
| 20 | L Vertebral | 34.94 | 0.013 | 0.019 | 4.2 | 4.72 |
| 22 | L Radial | 22.99 | 0.014 | 0.018 | 3.6 | 4.16 |
| 24 | L Interosseous | 531.50 | 0.0009 | 0.07 | 3.6 | 3.64 |
| 25 | L Ulnar II | 26.58 | 0.014 | 0.018 | 3.6 | 4.16 |
| 26 | Intercostals | 7.57 | 0.054 | 0.009 | 6.9 | 9.06 |
| 31 | Hepatic | 21.92 | 0.021 | 0.015 | 30.6 | 31.44 |
| 32 | Gastric | 3.84 | 0.033 | 0.012 | 9.3 | 10.62 |
| 33 | Splenic | 28.80 | 0.014 | 0.018 | 11.6 | 12.16 |
| 34 | Superior Mesenteric | 5.20 | 0.081 | 0.007 | 15.9 | 19.14 |
| 36 | R Renal | 5.82 | 0.068 | 0.008 | 5.9 | 8.62 |
| 38 | L Renal | 5.82 | 0.068 | 0.008 | 5.9 | 8.62 |
| 40 | Inferior Mesenteric | 41.50 | 0.011 | 0.02 | 11.9 | 12.34 |
| 43 | Inner Iliac | 31.70 | 0.014 | 0.018 | 3.5 | 4.06 |
| 45 | Deep Femoral | 18.05 | 0.023 | 0.014 | 14.4 | 15.32 |
| 47 | Anterior Tibial | 5.52 | 0.023 | 0.014 | 14.4 | 15.32 |
| 48 | Posterior Tibial | 41.09 | 0.01 | 0.021 | 14.4 | 14.80 |
| **0D Capillaries** | | | | | | |
| **LB** | - | 0.29 | 0.1 | 0.003 | 202.3 | 214.4 |
| **UB** | - | 0.97 | 0.03 | 0.003 | 74.8 | 79.3 |
| **0D Venules** | | | | | | |
| **LB** | - | 0.04 | 1.5 | 0.001 | 780.4 | 968.6 |
| **UB** | - | 0.14 | 0.5 | 0.001 | 288.6 | 358.3 |
| **0D Veins** | | | | | | |
| **LB** | - | 0.009 | 75 | 0.0005 | 1378.9 | 1919.3 |
| **UB** | - | 0.03 | 15 | 0.0005 | 323.5 | 450.2 |
| **0D Venae Cavae** | | | | | | |
| **LB** | - | 0.0005 | 15 | 0.0005 | 14.8 | 21.4 |
| **UB** | - | 0.0005 | 5 | 0.0005 | 4.9 | 7.1 |
| **0D Pulmonary** | | | | | | |
| **Arteries** | - | 0.08 | 3.8 | - | 44.3 | 59.3 |
| **Veins** | - | 0.01 | 20.5 | - | 232.8 | 311.2 |

**Supplementary Table 3.** 0D cardiac chambers and valves parameter settings (ra, rv, la and lv denote right atrium and ventricle, left atrium and ventricle, respectively, whereas tv, pv, mv, av refer to tricuspid, pulmonary, mitral and aortic valve, respectively).

| **0D Heart Chambers** | | | | |
| --- | --- | --- | --- | --- |
| **Parameter** | **ra** | **rv** | **la** | **lv** |
| E_A_ [mmHg/ml] | 0.06 | 0.65 | 0.07 | 2.75 |
| E_B_ [mmHg/ml] | 0.14 | 0.073 | 0.11 | 0.083 |
| V_un_ [ml] | 6 | 12 | 6 | 7 |
| V [ml] | 91.9 | 116.3 | 109.9 | 109.4 |
| t_ac_ [s] | 0.8 RR | - | 0.8 RR | - |
| T_ac_ [s] | 0.17 RR | - | 0.17 RR | - |
| t_ar_ [s] | t_ac_ + T_ac_ | - | t_ac_ + T_ac_ | - |
| T_ar_ [s] | T_ac_ | - | T_ac_ | - |
| T_vc_ [s] | - | 0.3 $\sqrt{\mathrm{RR}}$ | - | 0.3 $\sqrt{\mathrm{RR}}$ |
| T_vr_ [s] | - | 0.5 T_vc_ | - | 0.5 T_vc_ |
| **0D Heart Valves** | | | | |
| **Parameter** | **tv** | **pv** | **mv** | **av** |
| R_va_·10^-6^ [mmHg s/ml] | 4.5 | 4.5 | 4.5 | 4.5 |
| L_va_·10^-6^ [mmHg s/ml] | 3.75 | 3.75 | 3.75 | 3.75 |
| B_va_·10^-6^ [mmHg s^2^/ml^2^] | 4.8 | 5.67 | 4.8 | 5.67 |
| k_p,va_ [rad/(mmHg s^2^)] | 5500 | 5500 | 5500 | 5500 |
| k_q,va_ [rad/(ml s^2^)] | 2 | 2 | 2 | 2 |
| k_f,va_ [rad/(ml s^2^)] | 50 | 50 | 50 | 50 |
| k_v,va_ [rad/(ml s^2^)] | 3.5 | 3.5 | 3.5 | 3.5 |

**Supplementary Table 4.** Baroreflex control parameter settings.

| **Arterial Baroreflex** | | | | |
| --- | --- | --- | --- | --- |
| **y_m_** | **α_m_** | **β_m_** | **γ_m_** | **τ_m_** |
| HR/HR_0_ | 0.75 | 0.75 | 1 | 3 |
| E_rv/lv_/E_rv/lv0_ | 0.40 | - | 0.80 | 3 |
| R_art/cap_/R_art/cap0_ | 0.80 | - | 0.60 | 15 |
| C_ve/v_/C_ve/v0_ | -0.20 | - | 1.10 | 30 |
| V_un,ve/v_/V_un,ve/v0_ | -0.42 | - | 1.21 | 30 |

**Supplementary Table 5.** 0D Coronary microvascular districts parameter settings.

| **0D Coronary Microvasculature** | | | |
| --- | --- | --- | --- |
| **G** | **RV** | **S** | **LV** |
| Vessels #id | 59, 61 | 56, 62 | 52, 53, 54, 57 |
| W_G_ [hg] | 0.46 | 0.54 | 1.04 |
| R_0,jj,T,G_ [mmHg s/ml] | [2903 , 2364 , 2030] | [934 , 827 , 719] | [1048 , 927 , 806] |
| γ_jj_ | [0.31 , 0.33 , 0.36] | [0.31 , 0.33 , 0.36] | [0.31 , 0.33 , 0.36] |
| γ_CEP,jj_ | [0.2 , 0.6 , 1] p_rv_ | [p_rv_ , 0.5(p_rv_+p_lv_) , p_lv_] | [0.2 , 0.6 , 1] p_lv_ |
|  | | | |
| C_1,T_ [ml/(mmHg hg)] | 0.026 | V_0,1,T_ [ml/hg] | 2.5 |
| C_3,T_ [ml/(mmHg hg)] | 0.1270 | V_0,3,T_ [ml/hg] | 8 |
| - | - | φ_SIP_ [ml] | 8.9 |

**Supplementary Table 6.** Inter-frequency analysis comparing the steepness of curve defined by the three layer-specific $Q_{AF,b}$/$\bar{Q}_{SR}$ for each microvascular coronary district (myocardial layers conventionally indexed as EPI = 1, MID = 2, ENDO = 3).

| **Coronary microvascular district and ventricular rate** | **Slope coefficient** | **Standard error** | **Comparison** | **p-value** |
| --- | --- | --- | --- | --- |
| **LAD** | | | | |
| 75 bpm | -0.0068 | 0.0014 | 75 vs 100 bpm | 0.432 |
| 100 bpm | -0.0083 | 0.0013 | 100 vs 125 bpm | 0.446 |
| 125 bpm | -0.0097 | 0.0013 | 75 bpm vs 125 bpm | 0.129 |
| **LCx** | | | | |
| 75 bpm | -0.0068 | 0.0014 | 75 vs 100 bpm | 0.432 |
| 100 bpm | -0.0083 | 0.0013 | 100 vs 125 bpm | 0.429 |
| 125 bpm | -0.0097 | 0.0012 | 75 bpm vs 125 bpm | 0.116 |
| **RCA** | | | | |
| 75 bpm | -0.0015 | 0.0017 | 75 vs 100 bpm | 0.926 |
| 100 bpm | -0.0017 | 0.0013 | 100 vs 125 bpm | 1.000 |
| 125 bpm | -0.0017 | 0.00093 | 75 bpm vs 125 bpm | 0.918 |

LAD, left anterior descending artery; LCx, left circumflex artery; RCA, right coronary artery.

**Supplementary Table 7.** Summary statistics of mean aortic pressure, left ventricular end-diastolic pressure and right ventricular end-diastolic pressure across the different simulations.

| **Variable** | Mean aortic pressure [mmHg] | Left ventricular end-diastolic pressure [mmHg] | Right ventricular end-diastolic pressure [mmHg] |
| --- | --- | --- | --- |
| SR 75 | 92.71 ± 2.10 | 15.37 ± 0.18 | 10.61 ± 0.25 |
| AF 75 | 90.9 ± 6.29 | 15.73 ± 0.70 | 11.49 ± 0.75 |
| p-value | *< 0.001* | *< 0.001* | *< 0.001* |
| SR 100 | 97.20 ± 1.94 | 15.49 ± 0.22 | 9.18 ± 0.21 |
| AF 100 | 94.77 ± 4.92 | 15.78 ± 0.78 | 10.91 ± 1.07 |
| p-value | *< 0.001* | *< 0.001* | *< 0.001* |
| SR 125 | 100.03 ± 1.25 | 15.85 ± 0.22 | 9.82 ± 0.55 |
| AF 125 | 96.59 ± 3.44 | 16.10 ± 0.83 | 11.51 ± 1.21 |
| p-value | *< 0.001* | *< 0.001* | *< 0.001* |

LAD, left anterior descending artery; LCx, left circumflex artery; RCA, right coronary artery.

**Supplementary Figure 1.** RR beats for mean ventricular rates of 75, 100 and 125 bpm, respectively (from left to right). 2000 RR beats are extracted for each HR: (blue) SR, (red) AF. PDFs of the RR beats are reported in the inserts.


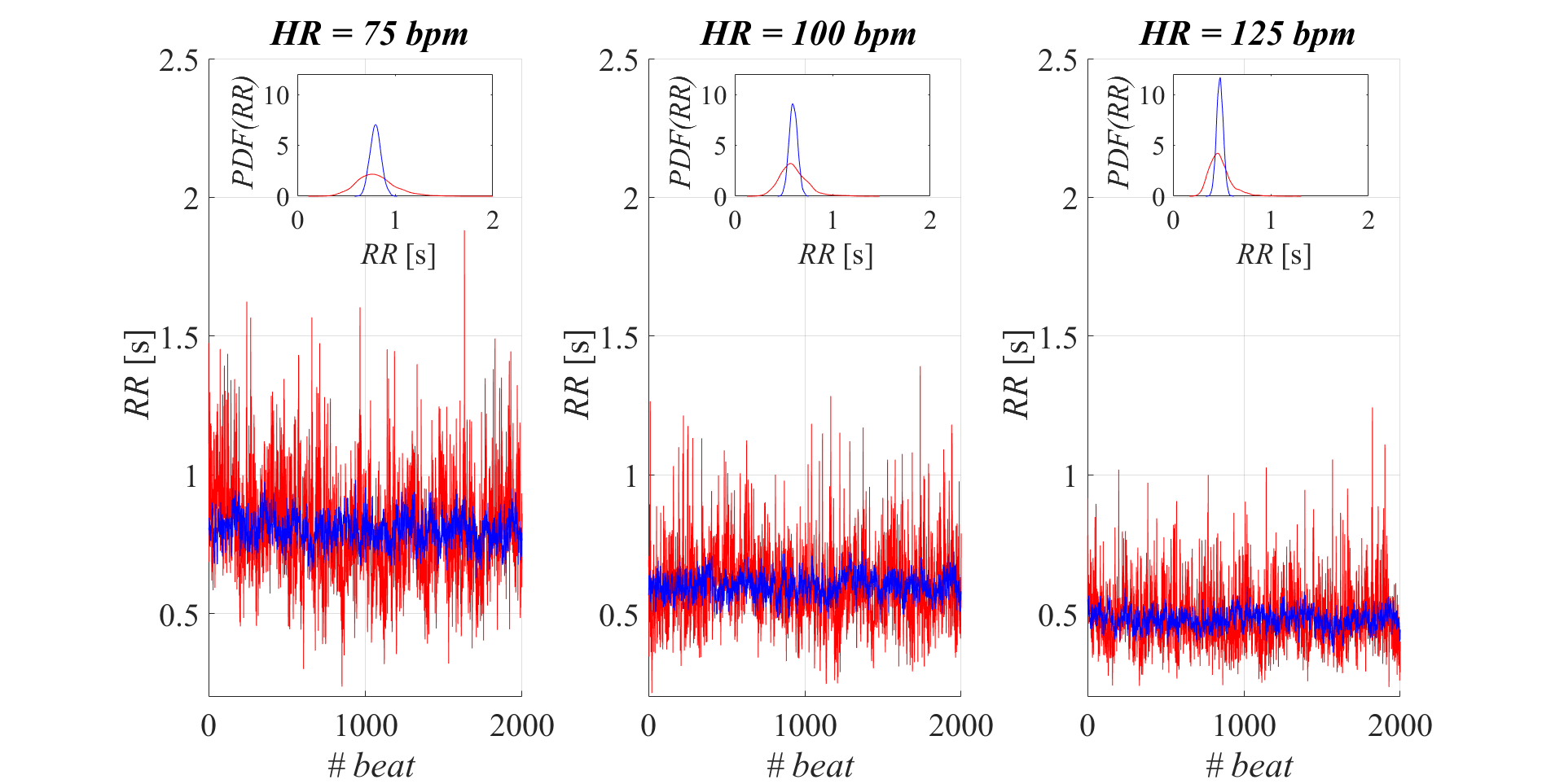


**Supplementary Figure 2.** Schematic illustration of the main input/output variables of the model (RR is the single beat duration, *i.e.,* 60/HR; *p, Q, V, A* and $\theta_{va}$*.*denote blood pressure, flow rate, volume, vessels cross section area, and valves opening angle, respectively).


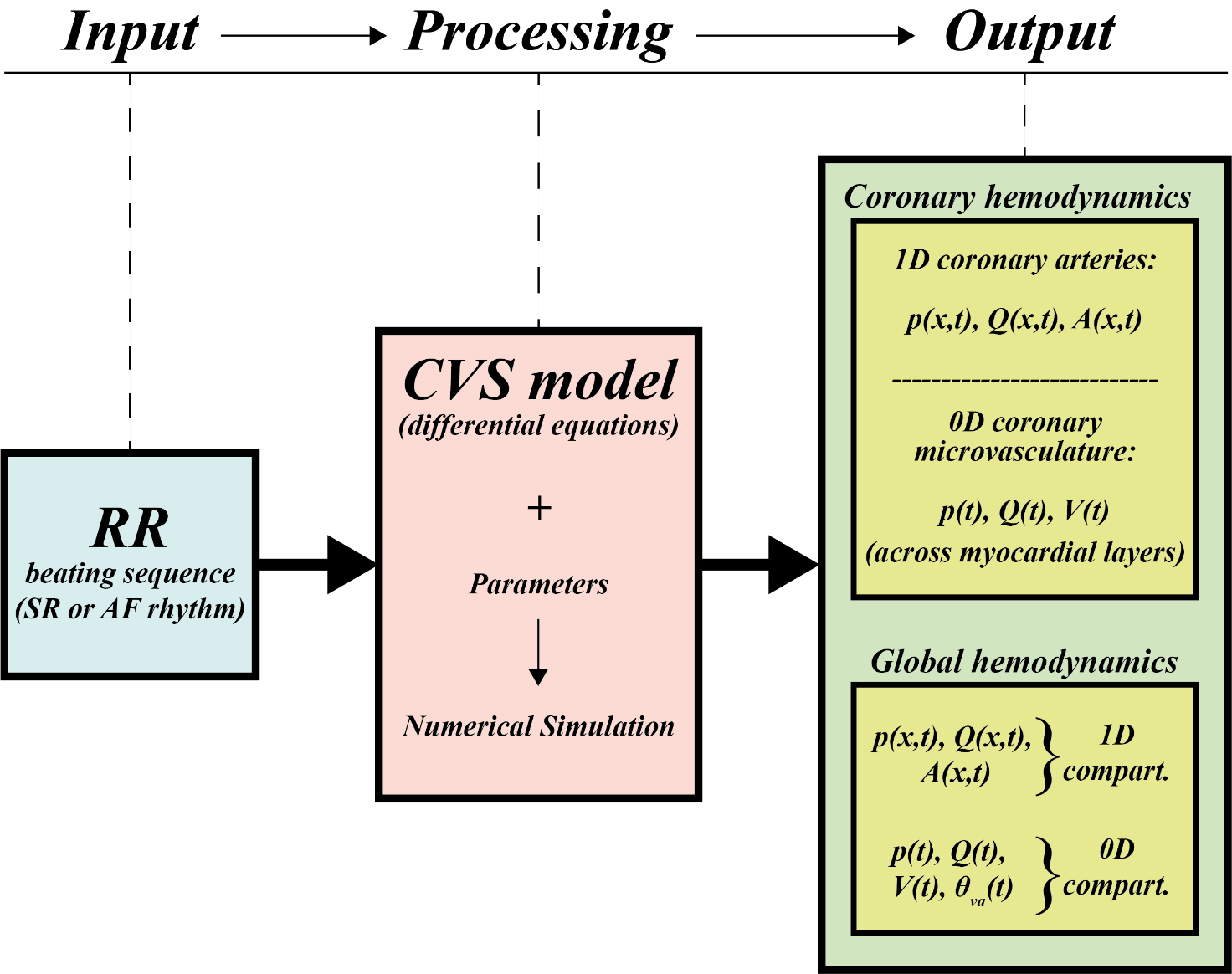

Supplement: Supplementary file 1 — Supplementary Information. [file 41598_2022_4897_MOESM1_ESM.docx]
